# The Impact of Maternal Obesity on NICU and Newborn Nursery Costs

**DOI:** 10.3389/fped.2022.863165

**Published:** 2022-05-18

**Authors:** Sharmeen Azher, Joaquim M. B. Pinheiro, Brendan Philbin, Jamie Gifford, Rubia Khalak

**Affiliations:** ^1^Albany Medical Center, Department of Pediatrics, Division of Neonatology, Albany, NY, United States; ^2^Albany Medical College, Department of Medical Education, Albany, NY, United States

**Keywords:** maternal obesity, neonate, cost impact, maternal obesity risk factors, NICU admission

## Abstract

**Background:**

Research on the effects of maternal obesity on neonates has focused on clinical outcomes. Despite growing interest in obesity as a driver of healthcare expenditure, the financial impact of maternal obesity in the neonatal setting is little understood.

**Objective:**

To determine if maternal obesity is associated with higher incurred costs in NICU and full-term nursery.

**Methods:**

Data for all live births (1/1/14–12/31/19) at our academic medical center was obtained from the New York State Perinatal Data System for infants >23 weeks gestational age. Financial data was obtained from the hospital's cos*t-*processing application. Infants with missing clinical and/or financial data were excluded. The NIH definition of obesity was used (BMI ≥ 30 kg/m^2^) to separate infants born to obese and non-obese mothers. Student's *t-*tests and chi square tests were used to compare maternal data, delivery, and infant outcomes between both groups. A logistic regression model was used to compare infant outcomes using odds ratios while controlling for maternal risk factors (smoking status, pre-pregnancy and gestational diabetes, pre-pregnancy and gestational hypertension). Multivariate regression analysis adjusting for maternal risk factors was also used to compare length-of-stay, total and direct costs in the NICU and full-term nursery between infant groups.

**Results:**

Of the 11,610 pregnancies in this retrospective study, obese mothers more frequently had other risk factors (smoke, pre-pregnancy and gestational diabetes, and pre-pregnancy and gestational hypertension). Infants born to obese mothers were more often preterm, had Cesarean delivery, lower APGAR scores, required assisted ventilation in the delivery room, and required NICU admission. Adjusting for maternal risk factors, infants born to obese mothers were less frequently preterm (OR 0.82 [0.74–0.91], *p* < 0.01) and had NICU stays (OR 0.98 [0.81–0.98], *p* = 0.02), but more frequently had Cesarean births (OR 1.54 [1.42–1.67], *p* < 0.01). They also had longer adjusted LOS (2.03 ± 1.51 vs. 1.92 ± 1.45 days, *p* < 0.01) and higher mean costs per infant in the full-term nursery ($3,638.34 ± $6,316.69 vs. $3,375.04 ± $4,994.18, *p* = 0.03) but not in NICU.

**Conclusions:**

Maternal obesity correlates with other risk factors. Prolonged maternal stay may explain increased LOS and costs in the full-term nursery for infants born to obese mothers, as infants wait to be discharged with mothers.

## Background

The rate of pre-pregnancy obesity in women has continued to increase across the United States, with nearly 3 in 10 women having obesity prior to becoming pregnant in 2019 (1). A plethora of literature documents the suboptimal outcomes that can arise for pregnant women with obesity and their infants. For expecting mothers, maternal obesity increases the risk of obstetrical outcomes such as gestational hypertension, gestational diabetes mellitus, pre-eclampsia, and Cesarean delivery (2–6). For their offspring, maternal obesity confers higher risk of prematurity, macrosomia, and infant death (7–11). Previous research has additionally shown that infants born to mothers with level III pre-pregnancy obesity are more likely to require delivery room resuscitation, assisted ventilation beyond 6 h of age, and NICU admission, which constitute more resource-intensive care (12).

A small but growing collection of studies examine the financial burden of obesity and related healthcare utilization. A 2016 meta-analysis based on 9 articles between January 2000 and September 2017 found that obesity accounted for nearly 32% of medical costs for affected individuals (13). For Canadian children born to mothers with pre-pregnancy obesity, Kuhle et al. (14) identified higher rates of healthcare utilization and costs over the first 18 years of life. Although the topic has been gaining more attention, maternal obesity has not been consistently linked with increased costs for offspring in the inpatient setting. Length-of-stay (LOS) is a key metric analyzed in cost studies. An earlier Australian study demonstrated that maternal obesity was associated with increases in LOS and costs related to maternal hospitalization but did not observe an increase in infant hospital costs (15). In a later study, Whiteman et al. (16) used a Florida-wide data set to demonstrate that infants born to mothers with obesity had higher inpatient costs during their first year of life compared with infants born to mothers without obesity. This effect was exacerbated by the presence of gestational diabetes mellitus, and consistent across all levels of obesity, per the National Institutes of Health definition of obesity as BMI ≥ 30 kg/m^2^ with further classification into levels I, II, and III. While comprehensive, a serious limitation of the Whiteman et al. study is the use of charge data to estimate direct medical costs. Charge data indicates what a hospital bills for services, but poorly represents the actual costs of rendered care. Our study explores whether maternal obesity is associated with greater costs of care in the neonatal period by using precise estimates of incurred costs at a tertiary care hospital.

## Methods

**Figure 1:**
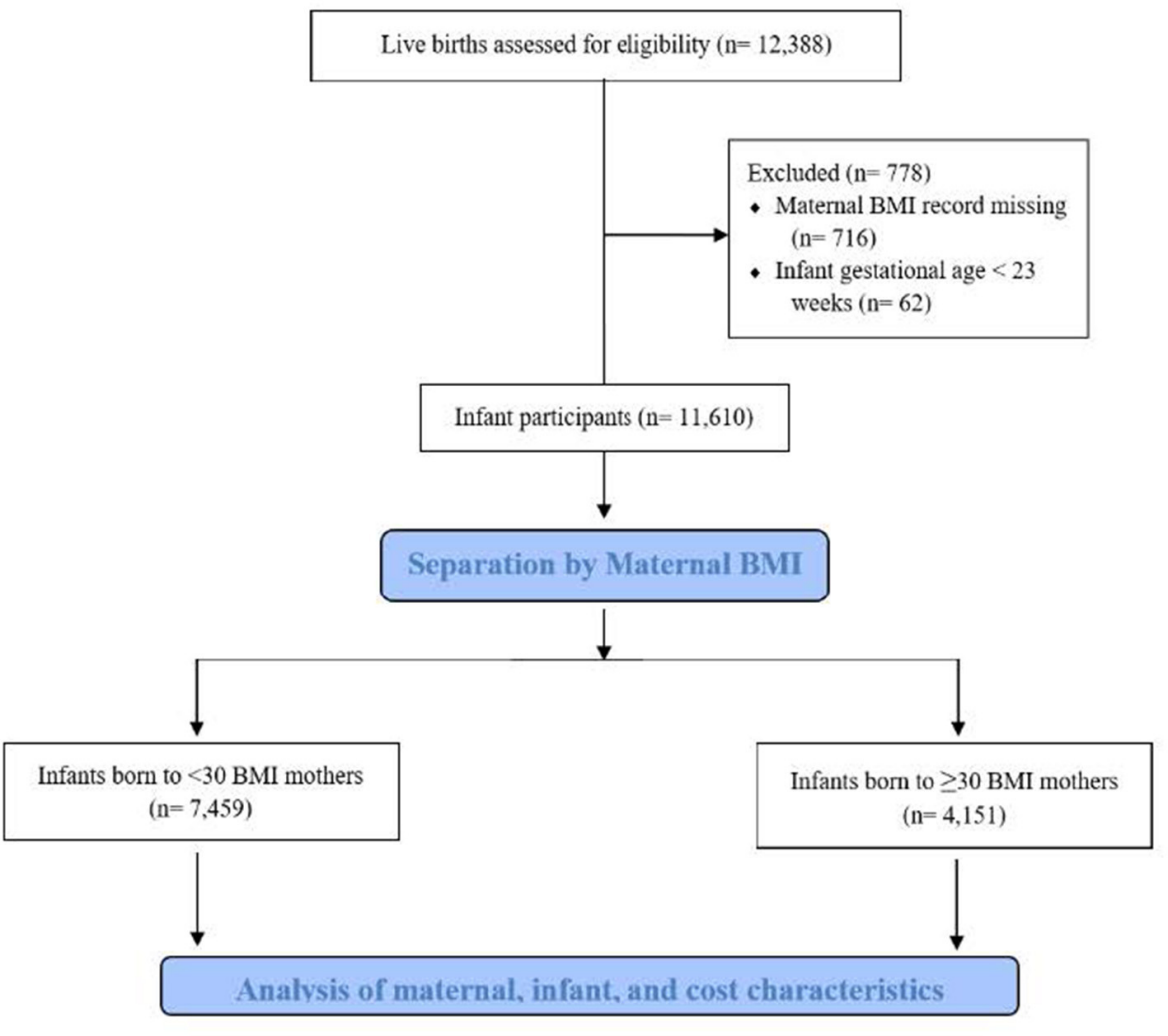
Consort diagram.

After study approval from our institution's Investigational Review Board, we obtained data for all live births from January 1, 2014, through December 31, 2019, at our academic medical center in eastern New York from the New York State Perinatal Data System, a database that contains mandated reporting on mothers and infants born in New York State hospitals. Mother-infant dyads with missing clinical data were excluded, as were any infants born at <23 weeks of gestational age (Figure 1). There were 574 sets of twins and 91 sets of triplets in the study. Twins were counted as two separate births having a unique mother; triplets were similarly treated. Pre-pregnancy BMI was measured in the first trimester for all participating mothers during their first clinic visit. Mothers with self-reported BMI or unknown BMI were excluded from the study. Obesity was defined as BMI ≥ 30 kg/m^2^, consistent with the National Institute of Health definition, and was not further classified into levels of obesity. The comparison study group consisted of infants born to mothers with BMI <30 kg/m^2^. For mothers, the following characteristics were collected: age at delivery, smoking status, gravidity, pre-pregnancy BMI, pre-pregnancy diabetes, gestational diabetes, pre-pregnancy hypertension, and gestational hypertension. Delivery characteristics and outcomes of infants in the study included pre-term birth, Cesarean delivery, gestational age, birth weight, APGAR scores, whether assisted ventilation was used in the delivery room setting, and whether the infant had a stay in our institution's level 4 NICU. Assisted ventilation included nasal continuous positive airway pressure and endotracheal ventilation. Prematurity was defined as <37 weeks gestational age.

Length of Stay (LOS) was calculated from our institution's electronic medical record (EMR) to the minute as the amount of time from birth hospitalization to discharge, including the day of discharge. Consequently, infants discharged on the day of birth are included in this study. Since the purpose of this study is to evaluate neonatal care, all LOS and cost data was restricted to the first 28 days after birth. If an infant had a hospital stay >28 days, LOS was deemed 28 days for the purpose of this study and only the costs of care rendered through the 28th day of life were assessed. If an infant was discharged home in the neonatal period but returned to the hospital prior to 28 days of life, clinical and financial data from this second visit were excluded. Infants requiring an additional hospital visit for common reasons such as sepsis rule out, inadequate feeding sequalae, and hyperbilirubinemia are generally cared for at lower-acuity institutions in our region, which feature a level 2 NICU. Our hospital, which features a level 4 NICU serves as the region's referral center for high-acuity presentations requiring intensive subspeciality care. Thus, including data in the neonatal period from hospital visits to our institution after birth would not be representative of more late-onset presentations seen in the study population.

Financial data for each infant were extracted from the hospital's internal cos*t-*processing application, which tabulates costs per individual based on services, supplies, medications, and other resources used by the patient. “Cost,” in this study, refers to the estimated dollar amount incurred by the hospital (not charges or revenue) to provide care to the patient based on facility Relative Value Units, excluding physician fees. Total costs consist of variable and fixed costs. Variable costs vary with changes in volume of either the patient or the resource under consideration (e.g., the staff and resources required to complete an ultrasound). Variable costs are measured by our institution only as “direct costs” and referred to as such in this study. Fixed costs do not change per volume of patient or resource to be used (e.g., the cost of an ultrasound machine). Total and direct costs were collected for each infant and further stratified into setting of care: NICU or newborn nursery. The newborn nursery is our institution's pos*t-*natal ward. Assessing costs per setting on days involving a transfer of care between the NICU and nursery proved challenging because our internal cos*t-*processing application records the costs of care for an infant by day and not by setting. On days in which a transfer of care occurred, an infant's cost by setting was estimated using duration of stay in each setting. For example, if an infant spent ¾ of the day in the NICU and ¼ of the day in the nursery, then ¾ of the day's total cost of care were distributed to the NICU and ¼ of the total cost was distributed to the nursery.

### Statistical Analysis

Student *t-*tests and chi square tests were used to compare normally distributed maternal characteristics between mothers with and without obesity. Similarly, student *t-*tests and chi square tests were used for a baseline comparison of delivery and infant outcomes between both study groups. A logistic regression model was used to compare selected infant outcomes while controlling for maternal risk factors (smoking status, pre-pregnancy and gestational diabetes, pre-pregnancy and gestational hypertension). These results were reported using adjusted odds ratios. Maternal age, which may influence delivery and infant outcomes, and parity, which may correlate with maternal age, were not found to significantly contribute to constructed regression models and were thus excluded. Multivariate regression analysis adjusting for above maternal risk factors was also used to compare length-of-stay, total and direct costs in the NICU and full-term nursery between infant groups. Statistical significance was established at the *p* < 0.05 level. Statistical analyses involving clinical data (Tables 1, 2) were performed using STATA software, Special Edition version 12.1 (2012, StataCorp, College Station, Texas). Statistical analyses involving financial data (Table 3) were performed using the R software (2020, R Core Team, Vienna, Austria).

**Table 1:** Characteristics of mothers without and with obesity.

	**Mothers with BMI <30 kg/m^**2**^ (*n =* 7,069)**	**Mothers with BMI ≥30 kg/m^**2**^ (*n =* 3,911)**	***p*-value**
Mean age in years	29.7	30.2	<0.01
Smokers	1,404 (19.8%)	965 (24.7%)	<0.01
Gravidity	2.73	3.16	<0.01
Pre-pregnancy BMI	24.1	37.8	<0.01
Pre-pregnancy diabetes	170 (2.4%)	223 (5.7%)	<0.01
Gestational diabetes	581 (8.2%)	749 (19.2%)	<0.01
Pre-pregnancy hypertension	230 (3.3%)	532 (13.6%)	<0.01
Gestational hypertension	881 (12.5%)	882 (22.6%)	<0.01

**Table 2:** Delivery characteristics and outcomes of infants born to mothers without and with obesity.

	**Infants born to Mothers with BMI <30 kg/m^**2**^ (*n =* 7,459)**	**Infants born to Mothers with BMI ≥30 kg/m^**2**^ (*n =* 4,151)**	***p*-value**	**Adjusted odds ratio [95% CI]**	***p*-value**
Pre-term birth	1,374 (18.4%)	827 (19.9%)	<0.05	0.82 [0.74, 0.91]	<0.01
Cesarean delivery	2,741 (36.8%)	2,145 (51.7%)	<0.01	1.54 [1.42, 1.67]	<0.01
Gestational age (weeks)	37.5 (3.1)	37.3 (3.1)	<0.01		
Mean birth weight in grams (± SD)	3,034.3 (770.9)	3,103.4 (824.2)	<0.01		
**Apgar score @ 5 mins**					
0–3	93 (1.3%)	51 (1.2%)	] <0.01		
4–6	314 (4.2%)	231 (5.6%)			
7–10	7,034 (94.3%)	3,845 (92.6%)			
Assisted ventilation in delivery room	1,384 (18.6%)	942 (22.7%)	<0.01	1.01 [0.91, 1.12]	0.85
NICU admission	1,625 (21.8%)	1,026 (24.7%)	<0.01	0.89 [0.81, 0.98]	0.02

**Table 3:** Length of stay (LOS) and cost characteristics of infants born to mothers without and with obesity after adjusting for smoking status, pre-pregnancy diabetes, gestational diabetes, pre-pregnancy hypertension, gestational hypertension.

	**Infants born to Mothers with BMI <30 BMI kg/m^**2**^ (*n =* 7,456)**	**Infants born to Mothers with BMI ≥30 kg/m^**2**^ (*n =* 4,151)**	***p*-value**
Total LOS in days (± SD)	5.4 (7.5)	5.8 (7.6)	0.40
NICU LOS in days (± SD)	3.5 (8.1)	3.8 (8.3)	0.86
Newborn Nursery LOS in days (± SD)	1.9 (1.5)	2.0 (1.5)	<0.01
Mean total cost per infant (± SD)	$12,290.10 ($21,543.39)	$13,372.86 ($22,240.12)	0.39
Mean direct cost per infant (± SD)	$5,351.62 ($10,204.08)	$5,802.89 ($10,626.56)	0.49
**Costs per infant by setting**			
**NICU**			
Mean total cost per infant (± SD)	$8,915.06 ($21,772.61)	$9,734.52 ($22,254.07)	0.76
Mean direct cost per infant (± SD)	$4,117.94 ($10,193.37)	$4,468.27 ($10,342.71)	0.83
**Full-term nursery**			
Mean total cost per infant (± SD)	$3,375.04 ($4,994.18)	$3,638.34 ($6,316.69)	0.03
Mean direct cost per infant (± SD)	$1233.68 ($2,264.87)	$1,334.62 ($3,271.44)	0.06

## Results

The final study population consisted of 11,610 mother-infant dyads. Of the 10,980 unique mothers under study, 35.6% had a BMI ≥ 30 kg/m^2^ while 64.4% had a BMI <30 kg/m^2^. Mothers with obesity were marginally older than their counterparts without obesity, more likely to smoke tobacco, and had a greater number of total pregnancies (Table 1). Mothers with obesity were also more likely to have pre-pregnancy diabetes, gestational diabetes, pre-pregnancy hypertension, and gestational hypertension than mothers with BMI <30 kg/m^2^. Dyads excluded due to missing financial information or more frequently, missing maternal BMI information from the first trimester, had higher incidence of maternal drug use during pregnancy and had smaller birth weights compared with the study population but were otherwise similar.

Table 2 compares delivery and other outcomes between infants in both study groups. Infants born to mothers with obesity were more likely to be pre-term than infants born to mothers without obesity, more likely to have a Cesarean birth, and had higher birth weights. However, adjusting for maternal risk factors (smoking status, pre-pregnancy diabetes, gestational diabetes, pre-pregnancy hypertension, and gestational hypertension), decreased the odds of pre-term birth for infants born to mothers with obesity (OR = 0.82, *p* < 0.01). After adjusting for these maternal risk factors, maternal obesity increased the odds of Cesarean delivery for infants (OR = 1.54, *p* < 0.01). There were small but statistically significant differences in APGAR scores at 5 min for babies in both groups, with infants born to mothers with obesity less likely to score in the 7 to 10 range. Infants born to mothers with obesity were also more likely to require assisted ventilation in the delivery room setting and more likely to require NICU admission. Adjusting for maternal risk factors decreased the odds of NICU admission (OR = 0.89, *p* = 0.02).

Three infants had missing financial data and were not included in the analysis of length-of-stay and cost characteristics (Table 3). Total LOS in the hospital within the neonatal period was comparable between both infant groups. While there was no difference in LOS in the NICU setting for both infant groups, infants born to mothers with obesity stayed slightly longer in the full-term nursery (2.0 days vs. 1.9 days, *p* < 0.01). Total and variable costs per neonate were comparable between both study groups. Stratifying these costs via setting of care delivered shows that in the NICU, there is no significant difference in mean total costs and mean variable costs between infants born to mothers with obesity compared with infants born to mothers without obesity. Infants born to mothers with obesity had $263.30 greater costs in the full-term newborn nursery compared with infants born to mothers without obesity (*p* = 0.03). Infants born to mothers with obesity also demonstrated higher costs in the newborn nursery by $100.94, but this was not a statistically significant finding.

## Discussion

We assessed the independent impact of maternal obesity on medical costs in the neonatal setting with regards to length-of-stay, total, and direct costs. Because the adverse effects of maternal obesity on neonatal outcomes are well-described in current literature and because the resulting care provided to infants with complications related to maternal obesity is more resource-intensive than for the healthy infant, we expected to observe an increase in length-of-stay for infants born to mothers with pre-pregnancy obesity as well as increased total and direct costs of care. To our surprise, we found no such difference in total LOS, total costs, or direct costs between infants born to mothers with pre-pregnancy obesity compared with infants born to mothers without obesity. This finding is consistent with a prior study's assertion that maternal obesity did not increase infant hospital costs during the birth admission (15).

There has been some work on cost analysis but primarily on the maternal hospital costs of maternal obesity without addressing infant morbidities and their healthcare costs. Vesco et al. evaluated the maternity-related costs of health care services in 750 women with severe maternal morbidity and compared to women without severe morbidity (17). Severe morbidity was defined as a life-threatening complication occurring during the delivery process. Although obesity may have been a factor for some of the women, this was not specifically considered. The research group of Morgan et al. estimated direct healthcare costs to the NHS of 224 women with obesity or overweight (18). They found that when compared to 260 normal BMI women, women with a BMI >25 kg/m^2^ were more likely to have increased healthcare usage and cost even when controlled for age, parity, ethnicity, and comorbidities. Although this study looked at both inpatient and outpatient costs to the mothers, infant morbidities and costs were not reviewed. Solmi and Morris used data from the Millennium Cohort Study to assess whether women with a BMI >25 kg/m^2^ had higher childbirth hospital costs (19). They found that compared to normal BMI women, women with BMI >25 kg/m^2^ had higher costs attributable to Cesarean delivery, pre-term delivery and increased length of stay. Watson et al. compared the economic costs, defined as hospital utilization and hospital costs for underweight, normal BMI and obesity BMI women (15). Their findings noted that maternal obesity increased length of stay and hospital costs when compared to normal BMI mothers. Again, the increased hospital utilization and medical costs of increased infant morbidities and prolonged infant stay were not reviewed in these studies.

On further stratification of LOS and cost metrics by setting of care during the neonatal period, we found that the mean cost of neonatal care was $263.30 more expensive for infants born to mothers with obesity in the full-term nursery, but costs were comparable in the NICU. With our finding of a difference in the incidence of prematurity for infants born to mothers with obesity and prematurity a major driver of NICU utilization, we expected the overall NICU costs to be higher. Maternal obesity has previously been linked with preterm birth, although current scholarship is more divided and emphasizes the interplay of maternal risk factors instead of maternal obesity alone (11, 20). It is less obvious why adjusting for maternal risk factors decreased the odds ratios for preterm birth and NICU admission in the setting of maternal obesity, suggesting that recognition of maternal obesity may counterbalance the effects of other risk factors. Further research is needed to understand how maternal obesity attenuates the pathophysiology of tobacco use, diabetes, and hypertension on the developing fetus.

That infants born to mothers with pre-pregnancy obesity have higher costs in the full-term nursery is likely related to our finding that such infants were shown to have a marginally longer LOS in the full-term nursery. The longer LOS of infants born to mothers with obesity is most likely explained by a significantly higher rate of Cesarean deliveries, which prolong LOS for mothers and by extension, infants, as healthy mothers and infants are discharged together at our institution. Higher rates of maternal complications may also help to explain our findings. Maternal obesity correlated with other maternal factors such as diabetes and hypertension, which have been identified in other literature as risk factors for maternal complications necessitating longer hospital stay. Furthermore, maternal obesity is an important risk factor for postpartum hemorrhage, among other obstetric complications (20). Although outside the scope of this study, which emphasized only the neonatal period, it is possible that the mothers with obesity in our study experienced complications that necessitated a longer hospital stay to recuperate from the physical stresses of birth, and their infants stayed longer in the full-term nursery as they waited to be discharged together. Watson et al. found that obese BMI was associated with marginally increased maternal LOS, which may provide further support for this hypothesis, although the authors do not speculate on the reason for their findings (15).

The high rate of Cesarean deliveries observed can likely be explained by our vast catchment area and by high rates of repeat Cesarean births. As our hospital is the tertiary referral center for more than 20 counties, women with significant comorbidities including level 3 obesity, gestational diabetes or hypertensive disorders, and identified fetal abnormalities, are sent to our hospital from smaller institutions for delivery. Also, most women with a prior Cesarean receive a repeat for their next delivery, with 86.2% of all Cesarean deliveries nationally attributable to repeat Cesareans (21). This trend, likely mirrored in our institution, may serve to compound the percentage of Cesarean births in our study.

Major strengths of our study include large sample size of mother-infant dyads obtained over a 5-year period, as well as its direct use of facility cost data rather than an approximation based on charge data. The retrospective nature of our study is also a strength as it minimized opportunities to influence cost of neonatal care during the study period. A limitation is that twin and triplet pregnancies were counted as different children to a unique mother, which may be a bias toward younger gestational age or smaller neonatal size. However, we would expect this to result in more obvious differences in cost and LOS between infants born to mother with normal BMI compared with infants born to mothers with obesity.

Our study is also limited by its use of BMI as a surrogate for obesity. Not only has BMI been called into question as a measure of obesity, but receiver operating characteristic (ROC) curve analysis suggests that based on percentage body fat, the traditional value of 30 kg/m^2^ may be too high a cutoff value to differentiate between individuals with and without obesity (22). Rather, Hart (22) suggests that a cutoff value closer to BMI 27 kg/m^2^ (currently in the NIH “overweight” BMI range) better captures individuals with obesity.

For this study, separating mother-infant dyads based on presence of maternal obesity was sufficient without the need for further stratification by level of obesity. Our previous research showed no difference in major reasons for NICU utilization, including the need for assisted ventilation beyond 6 h of birth when stratifying dyads according to level of maternal obesity and adjusting for maternal risk factors (23). Furthermore, our intent with this study was not to demonstrate a possible dose-response relationship between obesity levels and costs of care but perhaps examine this question with greater care in future work.

Our research contributes to the body of literature on the financial impact of maternal obesity in the neonatal setting and provides further motivation for health systems, especially in the United States, to engage in preventative care strategies.

## Data Availability Statement

The original contributions presented in the study are included in the article/supplementary material, further inquiries can be directed to the corresponding author/s.

## Author Contributions

RK, SA, and BP contributed to conception and design of the study. JG organized the database. SA, JG, and BP performed the statistical analysis. SA wrote the first draft of the manuscript. RK and JP wrote sections of the manuscript. All authors contributed to manuscript revision, read, and approved the submitted version.

## Funding

RK will be responsible for the publication fees with the assistance from her institution, Albany Medical Center, Division of Neonatology.

## Conflict of Interest

The authors declare that the research was conducted in the absence of any commercial or financial relationships that could be construed as a potential conflict of interest.

## Publisher's Note

All claims expressed in this article are solely those of the authors and do not necessarily represent those of their affiliated organizations, or those of the publisher, the editors and the reviewers. Any product that may be evaluated in this article, or claim that may be made by its manufacturer, is not guaranteed or endorsed by the publisher.

## References

[1] Driscoll AK Gregory ECW. Increases in prepregnancy obesity: United States, 2016-2019. NCHS Data Brief. (2020) 392:1–8. Available online at: http://europepmc.org/abstract/MED/33270551 33270551

[2] Aviram A Hod M Yogev Y. Maternal obesity: Implications for pregnancy outcome and long-term risks–a link to maternal nutrition. Int J of Gynecol Obstetr. (2011) 115:S6–10. 10.1016/S0020-7292(11)60004-0 22099446

[3] Ovesen P Steen R Ulrik K. Effect of prepregnancy maternal overweight and obesity on pregnancy outcome. Obstetr Gynecol. (2011) 118:305–12. 10.1097/AOG.0b013e3182245d49 21775846

[4] Magann EF Doherty DA Sandlin AT Chauhan SP Morrison JC. The effects of an increasing gradient of maternal obesity on pregnancy outcomes. Austral N Zeal J Obstetr and Gynaecol. (2013) 53:250–7. 10.1111/ajo.12047 23432797

[5] Wahabi HA Fayed AA Alzeidan RA Mandil AA. The independent effects of maternal obesity and gestational diabetes on the pregnancy outcomes. BMC Endo Dis. (2014) 14:1–7. 10.1186/1472-6823-14-47 24923207 PMC4065087

[6] Shin D Song WO. Prepregnancy body mass index is an independent risk factor for gestational hypertension, gestational diabetes, preterm labor, and small-and large-for-gestational-age infants. J Matern Fetal Neonat Med. (2015) 28:1679–86. 10.3109/14767058.2014.964675 25211384

[7] Gaudet L Ferraro ZM Wen SW Walker Mark. Maternal obesity and occurrence of fetal macrosomia: a systematic review and meta-analysis. BioMed Res Int. (2014) 2014:640291. 10.1155/2014/640291 25544943 PMC4273542

[8] Meehan S Beck CR Mair-Jenkins J Leonardi-Bee J Puleston R. Maternal obesity and infant mortality: a meta-analysis. Pediatr. (2014) 133:863–71. 10.1542/peds.2013-1480 24709933

[9] Santangeli L Sattar N Huda SS. Impact of maternal obesity on perinatal and childhood outcomes. B Pract Res Clin Obstetr Gynaecol. (2015) 29:438–48. 10.1016/j.bpobgyn.2014.10.009 25497183

[10] Bodnar LM Siminerio LL Himes KP Hutcheon JA Lash TL Parisi SM Abrams B. Maternal obesity and gestational weight gain are risk factors for infant death. Obes. (2016) 24: 490–8. 10.1002/oby.21335 26572932 PMC4731302

[11] Kong L Nilsson IAK Gissler M Lavebratt C. Associations of maternal diabetes and body mass index with offspring birth weight and prematurity. JAMA Pediatr. (2019) 173:371–8. 10.1001/jamapediatrics.2018.5541 30801637 PMC6450270

[12] Khalak R Cummings J Dexter S. Maternal obesity: significance on the preterm neonate. Int J Obes. 39:1433–6. 10.1038/ijo.2015.107 26051705

[13] Kim DD. Basu, A. Estimating the medical care costs of obesity in the United States: systematic review, meta-analysis, and empirical analysis. Val Heal. (2016) 19:602–13. 10.1016/j.jval.2016.02.008 27565277

[14] Kuhle S Muir A Woolcott CG Brown MM McDonald SD Abdolell M. Maternal pre-pregnancy obesity and health care utilization and costs in the offspring. Int J Obes. (2019) 43:735–43. 10.1038/s41366-018-0149-3 30006584 PMC6484728

[15] Watson M Howell S Johnston T Callaway L Khor S-L. Cornes, S. Pre-pregnancy BMI: Costs associated with maternal underweight and obesity in Queensland. Austral N Zeal J Obstetr Gynaecol. (2013) 53:243–9. 10.1111/ajo.12031 23316881

[16] Whiteman VE. Salemi, JL, Mejia De Grubb MC, Cain MA, Mogos MF, Zoorob RJ, Salihu HM. Additive effects of pre-pregnancy body mass index and gestational diabetes on health outcomes and costs. Obesity. (2015) 23:2299–308. 10.1002/oby.21222 26390841

[17] Vesco KK Ferrante S Chen Y Rhodes T Black CM. & Allen-Ramey, F. Costs of severe maternal morbidity during pregnancy in US Commercially Insured and Medicaid Populations: An observational study. Matern Child Health J. (2020) 24:30–8. 10.1007/s10995-019-02819-z 31655962

[18] Morgan KL. Obesity in pregnancy: a retrospective prevalence-based study on health service utilisation and costs on the NHS. BMJ Open 4.2. (2014) 4:e003983. 10.1136/bmjopen-2013-003983 24578535 PMC3939655

[19] Solmi F Morris S. Overweight and obese pre-pregnancy BMI is associated with higher hospital costs of childbirth in England. BMC Preg Child. (2018) 18:1–10. 10.1186/s12884-018-1893-z 29925340 PMC6011257

[20] Aly H Hammad T Nada A Mohamed M Bathgate S El-Mohandes A. Maternal obesity, associated complications and risk of prematurity. J Perinatol. (2010) 30:447–51. 10.1038/jp.2009.117 19693021

[21] Osterman MJK. Recent Trends in Vaginal Birth After Cesarean Delivery: United States, 2016–2018. Hyattsville, MD: National Center for Health Statistics NCHS Data Brief (2020) 359:1–8. 32487289

[22] Hart PD. Receiver operating characteristic (ROC) curve analysis: a tutorial using body mass index (BMI) as a measure of obesity. J Phys Act Res. (2016) 1:5–8.

[23] Khalak R Rijhsinghani A McCallum SE. Impact of maternal obesity on very preterm infants. Obes. (2017) 25:945–9. 10.1002/oby.21812 28332298

